# Drinking Water Treatment Using PES/ZnO Mixed Matrix Membranes: Enhanced Antifouling Performance and Rejection at Low Nanoparticle Loadings

**DOI:** 10.1021/acsomega.5c13032

**Published:** 2026-03-20

**Authors:** Viviane Bezerra Silva, José Carlos Mierzwa, Giovana Boaventura de Oliveira, Daiana Kotra Deda, Karine Cappuccio de Castro, Eduardo Lucas Subtil

**Affiliations:** † Laboratory of Urban Wastewater Treatment and Water Reuse (LabTAUS), Center of Engineering, Modeling and Applied Social Sciences, 74362 Federal University of ABC, Santo André, São Paulo 09210-170, Brazil; ‡ Polytechnic School, Department of Hydraulic and Environmental Engineering, University of Sao Paulo, Sao Paulo, 05508-220, Brazil; § 126786 SENAI Institute of Innovation in Advanced Materials and Nanocomposites, Rua Vitória Maria Médice Ramos, 330, São Bernardo Do Campo, São Paulo 09861-790, Brazil; ∥ School of Agricultural Sciences, 28108 São Paulo State University (UNESP), Botucatu, São Paulo 18610-307, Brazil

## Abstract

Poly­(ether sulfone) (PES) ultrafiltration mixed matrix membranes containing zinc oxide (ZnO) nanoparticles were fabricated and evaluated for drinking water treatment, with emphasis on fouling control and pollutant rejection. Unlike most studies that use model foulants, membranes were tested with organic-rich surface water from the Guarapiranga Reservoir, enabling a realistic assessment under drinking water conditions. Membranes (0–1.0 wt % ZnO) were prepared by nonsolvent-induced phase separation and characterized (SEM, AFM, porosity, hydrophilicity, zeta potential, permeability). Crossflow experiments with raw water included resistance partitioning, Hermia model analysis, and foulant extraction. Low ZnO loadings (0.25–0.50 wt %) delivered the best performance, reducing total fouling resistance by about 84% relative to pristine PES and achieving flux recovery above 96%. Improvements were linked to a more negative surface charge (−26 to −31 mV) and favorable pore structure that promoted electrostatic repulsion and reversible deposit formation. Membranes in this range also showed higher rejection of natural organic matter, with greater removal of color, TOC, and UV_254_ than both pristine PES and higher ZnO loadings. By contrast, the 0.75% ZnO membrane, despite its highest pure water permeability, exhibited greater irreversible fouling and lower rejection, while the 1.0% ZnO membrane behaved similarly to unmodified PES. Combining physicochemical characterization with real water tests, the study addresses practical and scale-up barriers to applying mixed matrix membranes. Findings indicate that PES-ZnO membranes with minimal nanoparticle loadings (0.25–0.50 wt %) offer a cost-effective and scalable strategy to improve flux stability, fouling control, and pollutant rejection in drinking water production.

## Graphical abstract

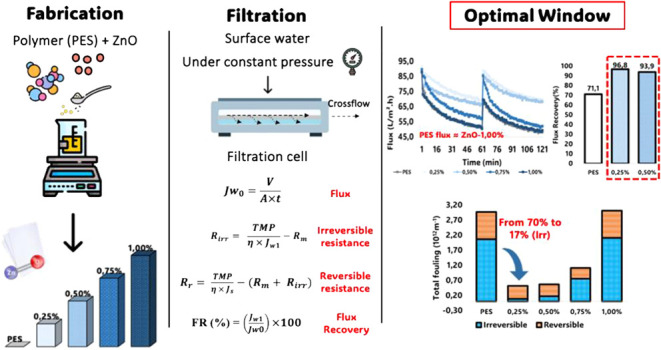

## 1 Introduction

In the current global context, one of the major challenges of rapid economic and social development is the sustainable exploitation of natural resources.^1^ Water pollution is considered one of the main causes of water scarcity, as the discharge of waste into streams and rivers is common and leads to degradation of water quality.^2,3^ This issue is further aggravated by climate change, which can increase the concentration of natural organic matter (NOM) into water bodies, raising total organic carbon (TOC) concentration in the water bodies used for public supply.^4–6^ As a result, conventional water treatment becomes more complex, demanding the incorporation of additional treatment steps for removing these organic compounds. In this context, water treatment using ultrafiltration (UF) membranes has emerged as a promising alternative to address these challenges.^7^

Particularly in urban areas, where physical space is limited and the impacts of diffuse pollution are more pronounced, UF membranes have been considered a feasible solution for the treatment of surface water sources.^8^ These membranes have proven effective in removing colloidal particles (turbidity), natural organic matter (NOM), microorganisms such as bacteria and viruses, as well as taste- and odor-related compounds.^9–14^ However, membrane performance can be significantly compromised by fouling, a complex process involving the accumulation of organic, inorganic, and biological materials on the membrane surface and within its pores, which reduces permeability, shortens membrane lifespan, and increases operational costs associated with maintenance and cleaning.^15–18^ This issue becomes even more critical in waters with high concentrations of dissolved and colloidal organic compounds, such as humic substances, proteins, and polysaccharides, whose presence has intensified due to increased TOC in many surface water bodies.^19^ In such cases, fouling tends to occur predominantly through adsorption and pore-blocking mechanisms,^20,21^ requiring the development of membranes with greater resistance to dissolved organic matter, especially in urban settings where water quality is affected by multiple diffuse pollution sources.

In light of these challenges, various fouling mitigation strategies have been investigated, particularly the development of mixed matrix membranes (MMMs), which incorporate nanomaterials into the polymer matrix.^22,23^ These additives can modify key membrane properties such as hydrophilicity, increasing the membrane’s negative surface charge, and pore structure, positively influencing hydraulic performance and resistance to deposit formation.^24–27^ Among the most studied nanomaterials are metal oxides such as TiO_2_, SiO_2_, Al_2_O_3_, and especially zinc oxide (ZnO), whose hydroxyl-rich surfaces (−OH) contribute to increasing membrane hydrophilicity and reducing hydrophobic interactions with organic compounds present in the water.^28,29^

Some studies have demonstrated that the incorporation of ZnO into UF membranes’ structure can lead to significant improvements, such as increased permeability, higher negative surface charge, and enhanced fouling resistance. Dipheko et al.^30^ focused on BSA (bovine serum albumin) fouling mitigation in ZnO/PES composite membranes, reporting improvements in both flux recovery and hydrophilicity. Vatanpour et al.^31^ explored the addition of ZnO to graphitic carbon nitride (g-C_3_N_4_) in UF membranes, demonstrating substantial improvements in water flux and contaminant rejection, including BSA and dyes, compared to unmodified membranes. Similarly, Dadashov et al.^32^ investigated the effects of different ZnO nanomaterial morphologies on the structure and performance of PES membranes, showing enhanced hydrophilicity and pure water flux with the use of ZnO nanorods.

Despite the growing interest in MMMs as an alternative to conventional polymeric membranes, important challenges still limit their practical application in drinking water systems, notably the lack of studies using real surface water matrices, which compromises the representativeness of the findings. Many investigations on MMM performance rely on model solutions, such as humic acid (HA) and BSA, which do not reflect the full physicochemical complexity and the foulants interactions of real water matrices. For instance, Lemos et al.^33^ observed markedly different behavior when using a humic solution to simulate the performance of graphene oxide-modified membranes for landfill leachate treatment. Similarly, membranes modified with graphene oxide (GO) and molybdenum disulfide (MoS_2_) exhibited superior permeability, fouling mitigation, and flux recovery when tested with model BSA and HA solutions.^34^ However, when the PES-MoS_2_ membrane was applied to real urban surface water, a significant shift in antifouling performance was observed, with reduced efficiency and a greater tendency for colloidal fouling, ultimately performing worse than the unmodified PES control membrane. In a study conducted by Hao et al.^35^ it was observed that the presence of metal ions, such as calcium (Ca^2+^) and iron (Fe^3+^), can considerably accelerate deposition by promoting the formation of larger humic acid aggregates, which then adhere more strongly to the membrane surface. Sioutopoulos et al.^36^ observed that the interaction between polysaccharides and proteins causes a higher propensity for deposition compared to a sample containing only one type of contaminant. These findings highlight that data obtained from simplified model solutions may overestimate MMM performance, masking key fouling mechanisms and potentially leading to misjudgments in real system design. Advancing studies with natural waters is therefore essential to the transition of MMMs from promising lab-scale innovations to viable technologies for large-scale drinking water treatment systems.

In contrast to previous PES/ZnO mixed matrix membrane studies largely based on model foulants or simplified feed waters, this work provides a systematic evaluation of low ZnO loadings under real surface water conditions, explicitly linking membrane physicochemical properties, fouling mechanisms, fouling reversibility, and pollutant rejection. In this context, the present study aimed to evaluate the influence of incorporating different proportions of zinc oxide (ZnO) nanoparticles into poly­(ether sulfone) (PES) ultrafiltration membranes, focusing on the physicochemical characterization of the membranes and their performance in the treatment of real surface water. The membranes were fabricated using the nonsolvent-induced phase inversion (NIPS) method and characterized in terms of morphology, porosity, hydrophilicity, zeta potential, and permeability. Subsequently, the membranes were tested in a crossflow filtration cell with surface water from an urban reservoir used for public supply, with the objective of assessing fouling formation, considering hydraulic resistances, and flux recovery. By systematically evaluating the performance of PES/ZnO membranes in treating real surface water, this study provides valuable insights to support the advancement of MMMs under more challenging and representative operational conditions, an important step toward their full-scale implementation for drinking water treatment.

## 2 Materials and Methods

### 2.1 Materials

Poly­(ether sulfone) (PES, 63,000 g·mol^–1^, Solvay) was used as the base polymer for the synthesis of all membranes, and *N*-methyl-2-pyrrolidone (NMP, 99.5%, Labsynth) served as the organic solvent for the preparation of the polymer solutions. Zinc oxide (ZnO) nanoparticles were synthesized using zinc acetate dihydrate (ZnC_4_H_6_O_4_, Synth) or zinc nitrate (Audaz Brasil) as the precursor, with a calcination time of 2 to 5 h at 250, 500, or 750 °C, for incorporation into the nanocomposite membranes. Isopropyl alcohol (C_3_H_8_O, 99.5%, Labsynth) was used during the membrane drying process.

### 2.2 Synthesis of ZnO Nanoparticles

ZnO nanoparticles were synthesized using two different precursors: zinc acetate and zinc nitrate, in order to evaluate the influence of the precursor on the final properties of the material. Sodium hydroxide (NaOH, PA-ACS-CRQ) was used as the precipitating agent, while isopropyl alcohol (PA-CRQ) was used for washing and dispersion. The following procedure refers to the synthesis based on zinc acetate, which yielded nanoparticles with the most suitable physicochemical characteristics for membrane application. Initially, 2.5 g of zinc acetate were dissolved in 100 mL of distilled water under stirring and heating until the solution reached 90 °C. Then, 7 g of NaOH solution (5 mol L^–1^) were added dropwise, and the mixture was kept under constant agitation and temperature for 10 min. The resulting precipitate was collected by centrifugation (5000  rpm, 10 min), washed three times with distilled water, and redispersed in 10 mL of isopropyl alcohol using an ultrasonic bath for 10 min. Subsequently, the ZnO nanoparticles were washed three additional times with isopropanol heated to 60 °C and dried in an oven at 60 °C for 24 h. The dried product was then calcined at different temperatures (250 °C, 500 °C, and 750 °C) and durations (2 and 5 h) to tune its crystallinity and stability.

For the synthesis based on zinc nitrate, a 0.71 mol·L^–1^ Zn­(NO_3_)_2_ solution was prepared by dissolving 12 g of Zn­(NO_3_)_2_ in 100 mL of distilled water under continuous magnetic stirring for 25 min. Separately, a 2.67 mol·L^–1^ NaOH solution was obtained by dissolving 3.2 g of NaOH in 30 mL of distilled water. The alkaline solution was added dropwise to the zinc nitrate solution, maintained at 70 °C under constant stirring, to promote homogeneous nucleation. The reaction system was kept under these conditions for 2 h. Upon completion of the reaction, the resulting suspension was cooled to room temperature and subjected to centrifugation at 7000 rpm for 10 min. The precipitate was washed three times with distilled water followed by three successive washes with isopropanol, and subsequently dried in a conventional oven at 160 °C for 3.5 h. For the calcination step, the same parameters used for materials obtained from zinc acetate were evaluated.

The ZnO sample synthesized from zinc acetate and calcined at 250 °C for 5 h was selected for membrane fabrication based on its favorable structural and colloidal properties. Moderate calcination temperatures have been shown to influence the structural and optical properties of ZnO nanoparticles by enhancing crystallinity and phase purity while limiting excessive grain growth and particle coalescence that occur at higher temperatures, which generally leads to increased particle size and reduced specific surface area in metal oxide nanomaterials.^37,38^

From a colloidal perspective, oxide nanoparticles interact differently with polar aprotic solvents depending on their surface energy and aggregation state, and stable dispersions of metal oxide nanostructures such as ZnO in polar solvents have been reported in the literature.^39^ In this work, the ZnO sample calcined at 250 °C for 5 h exhibited the smallest hydrodynamic diameter in aqueous dispersion (404.8 ± 145.5 nm), a relatively narrow size distribution in *N*-methyl-2-pyrrolidone (860.2 ± 196.7 nm), a high zeta potential (29.1 ± 6.99 mV), and absence of visible agglomerates, indicating enhanced colloidal stability. The combination of controlled thermal treatment and favorable dispersion behavior in NMP supports a more homogeneous distribution of ZnO nanoparticles within the polymer matrix during membrane casting, contributing to improved membrane morphology and performance.

### 2.3 Experimental Setup

Water sampling for fouling assessment was conducted at the Guarapiranga Reservoir (GR), one of the main drinking water sources for the Metropolitan Region of São Paulo (MRSP), located at coordinates 23°40′17″S–46°43′39″W. The raw water displays typical characteristics of surface sources with low turbidity, averaging around 7 NTU, apparent color ranging from 23 to 57 Pt–Co, and total organic carbon (TOC) concentrations between 6 and 12 mg L^–1^. The presence of dissolved organic matter was further evidenced by UV_254_ absorbance values ranging from 0.11 to 0.17 cm^–1^. Despite the low turbidity, the elevated levels of TOC and UV_254_ indicate a high potential for organic fouling in membrane filtration systems. As part of efforts to improve the water treatment performance at this reservoir, a membrane ultrafiltration module was installed at the Alto da Boa Vista Water Treatment Plant, which has been in operation since 2014–2015 and currently treats approximately 2 m^3^ s^–1^ of raw water.

Following the synthesis of the nanoparticles and the collection of surface water samples, the study was conducted in two main stages. In Stage I, control and nanocomposite membranes with varied ZnO contents were synthesized and characterized. In Stage II, the performance of the membranes was evaluated using real surface water. The filtration tests were conducted using a crossflow filtration system, as illustrated in Figure 1. The system consists of a tangential flow filtration cell with an effective membrane area of 85.1 cm^2^, a feed tank, and a peristaltic pump. The cell has two outlets: one for the permeate and another for the concentrated. The feed pressure was controlled using a needle valve located in the retentate line, and the transmembrane pressure (TMP) was calculated as the average of the pressures recorded by two manometers. The permeate was collected in a beaker and its volume was measured using an analytical balance (ATX224, Shimadzu, Japan; ±0.1 mg). All data were recorded and processed using a computer. All the experiments were performed at room temperature and data normalized for 20 °C.

**Figure 1:**
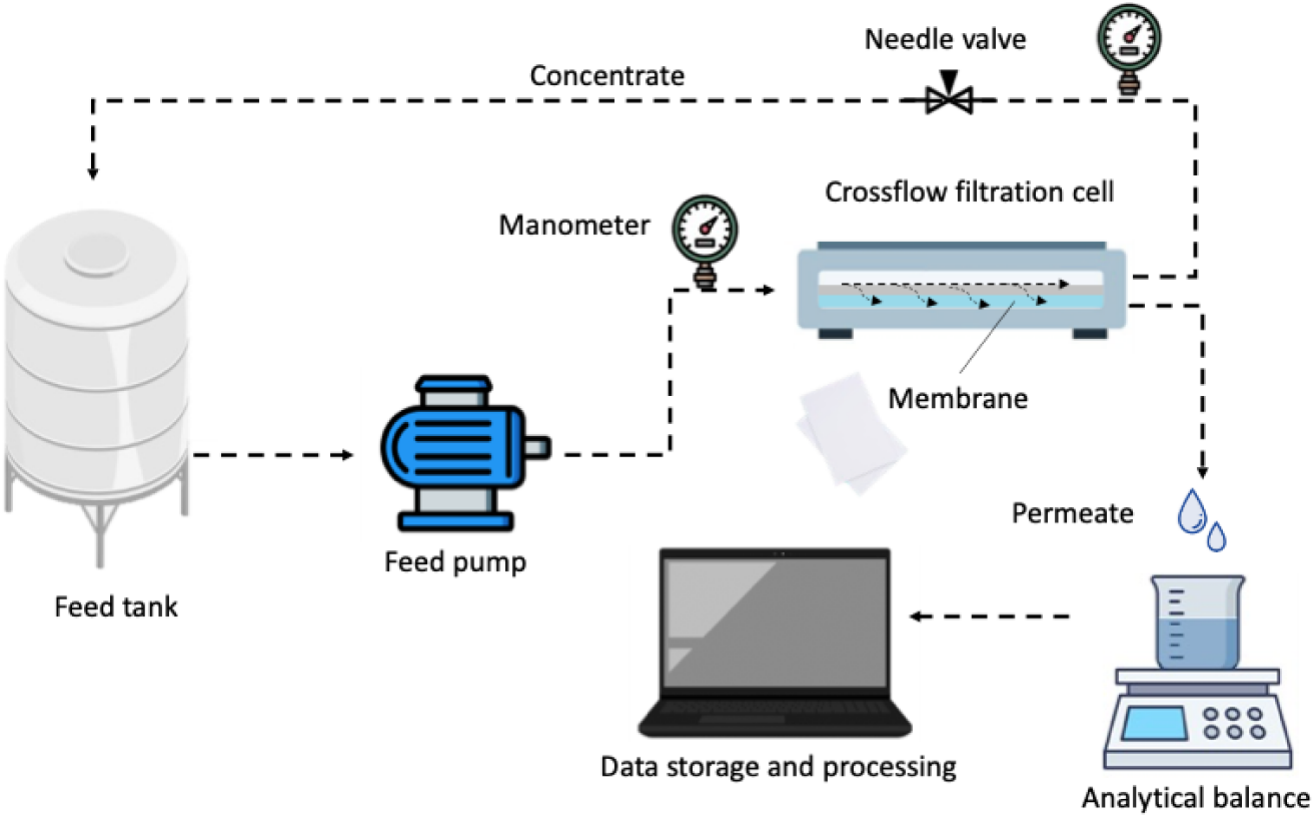
Schematic diagram of the crossflow filtration system.

### 2.4 Membrane Synthesis and Characterization

All membranes were synthesized using a fixed NMP concentration (83 wt %) and varying amounts of PES and ZnO nanoparticles, depending on the target composition. The control membrane was composed only of PES (17 wt %), while the nanocomposite membranes were prepared as follows: ZnO–0.25% (PES: 16.958 g/ZnO: 0.042 g), ZnO–0.50% (PES: 16.916 g/ZnO: 0.084 g), ZnO–0.75% (PES: 16.874 g/ZnO: 0.126 g), and ZnO–1.00% (PES: 16.832 g/ZnO: 0.168 g). The ZnO loading range (0.25–1.0 wt %) was selected in line with previous PES/ZnO mixed matrix membrane studies, where ZnO contents up to ∼2 wt % have been used to improve hydrophilicity and antifouling performance without compromising membrane formation.^40,41^ However, in the present study the upper limit was restricted to 1.0 wt % to ensure solution processability and to maintain economic feasibility, as higher nanoparticle loadings tend to increase material costs and casting complexity with limited additional performance benefits.

The membrane synthesis procedures followed the protocols described by Lemos et al.^33^ and Ragio et al.^34^ For polymer solution preparation, the materials were weighed using an analytical balance (ATX224, Shimadzu, Japan; ±0.1 mg). NMP was placed in a beaker under mechanical stirring, and PES was gradually added to ensure complete dissolution. The mixture was stirred for 24 h and then stored in a vacuum desiccator for an additional 24 h. Before casting, the solution was degassed in an ultrasonic bath for at least 1 h to remove air bubbles.

The membranes were fabricated via NIPS method. The polymer solutions were cast onto glass plates using a manual film applicator (Doctor Blade) at a constant speed, with the blade gap adjusted to 140 μm. Immediately after casting, the plate was immersed in a coagulation bath containing deionized water for 5 min. The membranes were then transferred to a second deionized water bath for 24 h to remove residual solvent and stored in deionized water until use. A schematic of the membrane casting steps is shown in Figure 2.

**Figure 2:**
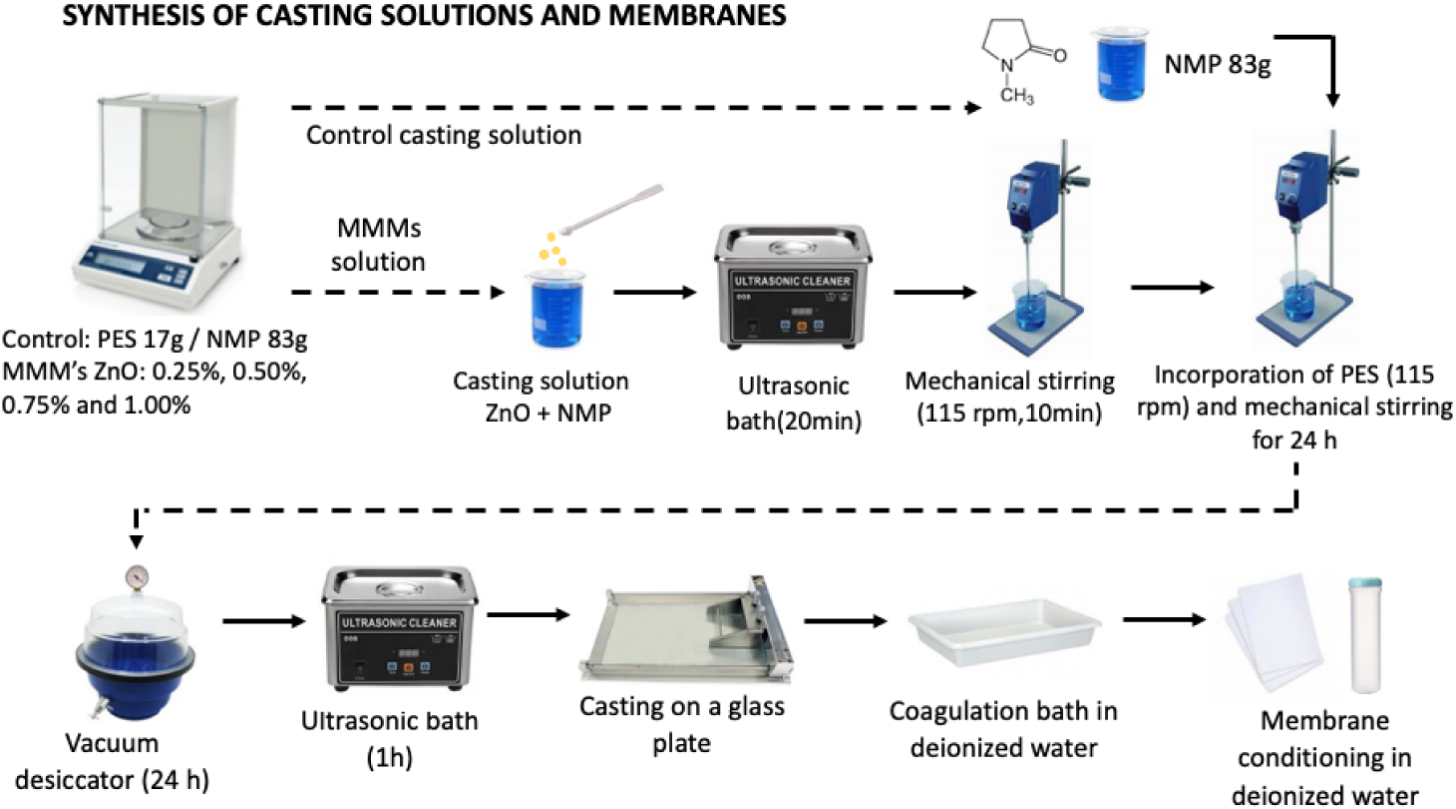
Schematic illustration of the membrane synthesis process.

For composition, morphology, contact angle, and zeta potential analyses, the membranes were subjected to a drying process involving their immersion in isopropyl alcohol for 24 h followed by drying on absorbent paper for another 24 h. This procedure was employed to preserve the pore structure integrity. Membrane morphology was assessed via scanning electron microscopy (SEM) using a compact JSM-6010LA microscope (JEOL) on membrane cross sections fractured in liquid nitrogen. Two samples were prepared for each membrane type. Surface roughness was analyzed via atomic force microscopy (AFM/SPM 5500, Agilent) using four samples per membrane type. Hydrophilicity was evaluated using a contact angle goniometer (SL150, Kino). Membrane samples were affixed to glass slides, and three measurements were taken on each of four samples per membrane type. Electrokinetic properties such as surface charge, particle repulsion, dispersion stability, and fouling propensity were assessed using zeta potential analysis (SurPASS3, Anton Paar), with two measurements performed for each membrane type.

Apparent porosity (ε) was estimated using the gravimetric method (also known as the wet–dry method), as described by Sherugar et al.^42^ First, membrane thickness (L, in m) and area (A, in m^2^) were measured. The average thickness was calculated from five measurements at different points using a digital micrometer (Digimess, 0–25 mm, ±0.002 mm). Five samples were used per membrane type. Each sample, previously soaked in distilled water, was gently blotted with tissue paper to remove surface water, and the wet mass (M_1_, in g) was determined using an analytical balance. The samples were then dried, and the dry mass (M_2_, in g) was recorded. Porosity was calculated using eq 1, where ρw is the density of water (g m^–3^).

1 $$\varepsilon \left(\%\right)=\frac{M_{1}-M_{2}}{\rho_{w}\times A\times L}\times 100$$

From the porosity and water flux data, the Guerout–Elford–Ferry equation (eq 2) was used to estimate the average pore radius (r_m_), where Q is the water flow rate (m^3^·s^–1^), η is the viscosity of water (Pa·s) at 25 °C, ΔP is the operating pressure (Pa), and l is the membrane thickness (m).

2 $$r_{m}=\sqrt{\frac{\left(2.9-1.75\varepsilon \right)\times 8\eta lQ}{\varepsilon \times A\times \Delta P}}$$

### 2.5 Hydraulic Characterization of the Synthesized Membranes

For the pure water flux (PWF) tests, the crossflow filtration cell (Figure 2) was fed with deionized water. For each membrane type, four to six PWF tests were performed. Permeate volume data were recorded every 2 min during the first 20 min, and every 5 min thereafter, up to a total of 120 min and 30 volume measurements. The transmembrane pressure (TMP) was maintained between 0.65 and 0.70 bar. After data collection, the PWF (L·m^–2^·h^–1^) at a steady state condition was calculated using eq 3, where V is the volume of permeate (L), A is the membrane area (m^2^), and t is the collection time (h).

3 $$F_{W}=\frac{V}{A\times t}$$

The hydraulic permeability (P_m_) was then calculated using eq 4, where p is the applied pressure (bar). The compaction factor (CF) was calculated as the ratio between the initial and final PWF values under steady-state conditions, as shown in eq 5.

4 $$P_{m}=\frac{F_{w}}{P}$$

5 $$\mathrm{CF}=\frac{{\mathrm{PWF}}_{\;\mathrm{initial}}}{{\mathrm{PWF}}_{\mathrm{final}}}$$

### 2.6 Fouling Propensity Analysis

Before starting the fouling tests with surface water, a chemical cleaning procedure was performed to remove any biological or organic matter that may have developed during membrane storage. The membranes were immersed for 30 min in a 100 mg·L^–1^ sodium hypochlorite (NaClO) solution, adjusted to pH 12 using sodium hydroxide (NaOH). Following the cleaning step, the membranes was submitted to compaction with deionized water for 60 min to further obtain the initial pure water flux (Jw_0_).

Each membrane underwent two filtration cycles using surface water. Each cycle consisted of a 60 min sample filtration (J_s_), during which permeate was collected every 1 min, followed by a 15 min recirculation of deionized water with the system depressurized and without permeation, and finally a 10 min deionized water filtration, also with permeate collection every 1 min (Jw_1_). The mass of the permeate was measured using an analytical balance (ATX224, Shimadzu, Japan; ±0.1 mg), and the corresponding volume was determined based on the density of water (998 kg m^–3^ at 20 °C).

This methodology was adapted from Ragio et al.^43^ After the fouling tests, the initial flux Jw_0_ was calculated using eq 6, where V is the permeate volume (L), A is the membrane area (m^2^), and t is the time interval between measurements (h).

6 $$Jw_{0}=\frac{V}{A\times t}$$

The intrinsic membrane resistance (R_m_) was calculated according to eq 7, where TMP is the transmembrane pressure (bar), Jw_0_ is the initial steady-state pure water flux (L m^–2^ h^–1^), and η is the dynamic viscosity of the water (Pa·s). The irreversible (R_irr_) and reversible (R_r_) resistances were determined using eqs 7 and 8, respectively. Furthermore, flux recovery ratio (RF) was estimated according to eq 9.

7 $$R_{\mathrm{irr}}=\frac{\mathrm{TMP}}{\eta \times J_{w1}}-R_{m}$$

8 $$R_{r}=\frac{\mathrm{TMP}}{\eta \times J_{s}}-\left(R_{m}+R_{irr}\right)$$

9 $$\mathrm{RF}=\left(\frac{J_{w1}}{J_{w0}}\right)\times 100$$

To understand the fouling mechanisms during the filtration of raw water using the different synthesized membranes, the classical Hermia model was applied. This model is widely applied to describe the flux decline behavior in membrane filtration systems and considers different blocking modes associated with particle deposition on or within the membrane structure.^43,44^ The four modes include complete blocking (n = 2), intermediate blocking (n = 1), standard blocking (n = 1.5), and cake layer formation (n = 0), each represented by a specific equation relating permeate flux to filtration time. Experimental flux data were fitted to the corresponding equations by minimizing the mean squared error (MSE) between the measured and predicted flux values. To enable a comparative analysis among the models, the inverse of the MSE values (1/MSE) was calculated and subsequently normalized on a scale from 0 to 1 within each model, where values closer to 1 indicate a better fit. This approach allowed for the identification of the predominant fouling mechanism under each evaluated condition.

### 2.7 Characterization of Foulant Substances

The amounts of proteins and humic substances accumulated on the membrane surface were evaluated as described by Subtil et al.^23^ and Ragio et al.^43^ A fouled membrane segment measuring 2 × 11 cm was cut into smaller pieces and placed in a 50 mL beaker containing 25 mL of deionized water. The sample was stirred using a magnetic stirrer for 4 min. After stirring, the solution was collected and analyzed for humic substances and protein content using the method of Lowry et al.^45^

### 2.8 Physicochemical Analyses

Physicochemical analyses of the samples collected during the filtration tests included measurements of color and turbidity using a colorimeter (Model HI83399) and a turbidimeter (Model HI98703), both from Hanna Instruments. Humic substances were quantified by UV_254_ absorbance following Standard Method 5910 B.^46^ Protein content was determined using the Lowry method^45^ and total organic carbon (TOC) was measured with a TOC-L analyzer (Shimadzu, Japan).

## 3 Results and Discussion

### 3.1 Zinc Oxide Nanoparticle Characterization (ZnO NPs)

The multifaceted characterization of zinc oxide nanoparticles (ZnO NPs) revealed the crucial influence of synthesis parameters, particularly the precursor salt and the dispersion medium, on the final properties of the particles. As shown in Figure 3, the DLS profiles clearly demonstrate that the chemical nature of the zinc precursor affects particle size distribution. Nanoparticles synthesized from zinc acetate (Figure 3A and C) exhibited narrower size distributions compared to those obtained from zinc nitrate (Figure 3B and D). This suggests that acetate ions may have favored more uniform nucleation and growth under the experimental conditions, an observation consistent with previous studies.^47^

**Figure 3:**
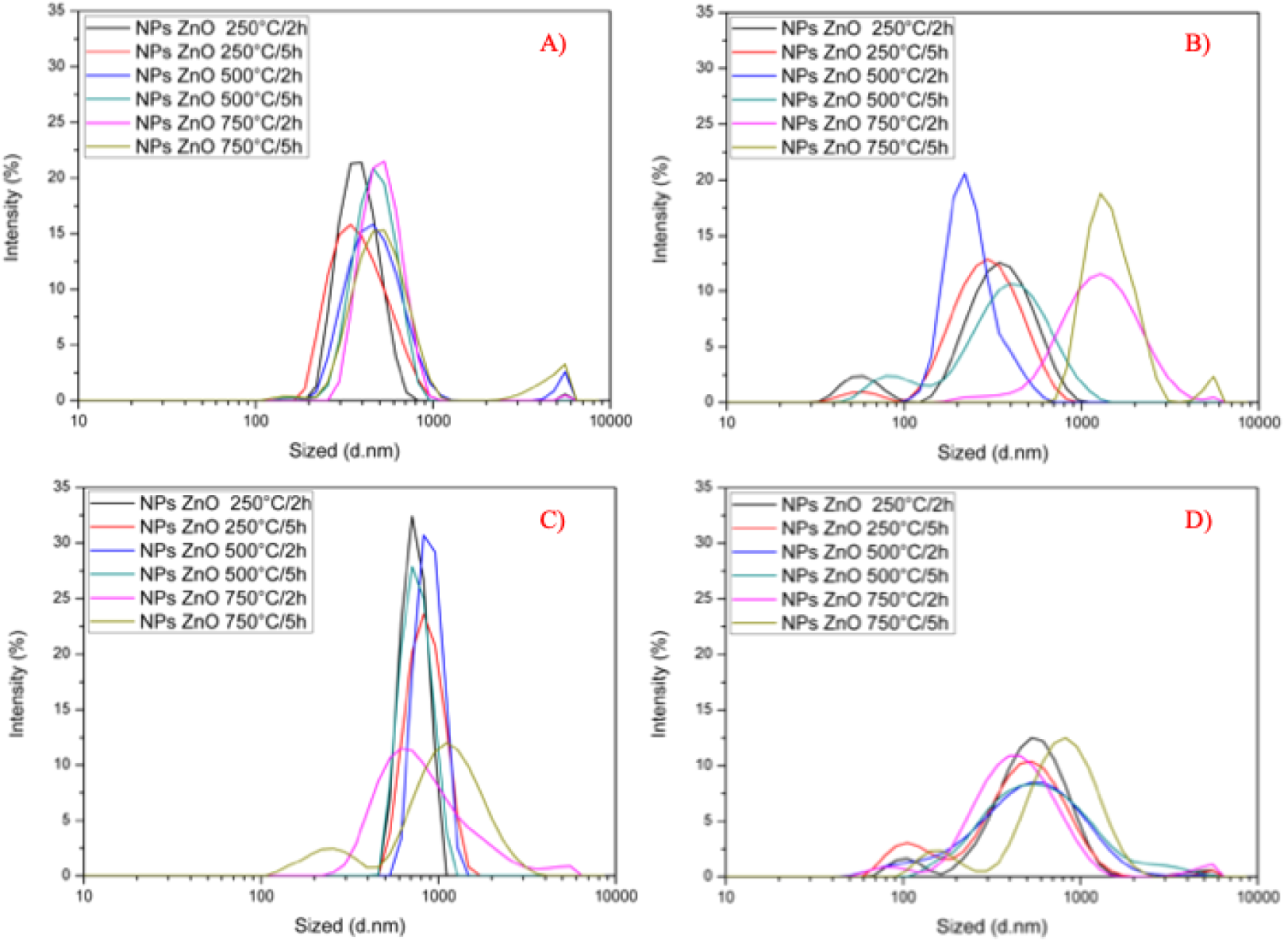
DLS profiles of ZnO nanoparticles synthesized from zinc acetate and zinc nitrate, dispersed in water and NMP: (A) zinc acetate in water, (B) zinc nitrate in water, (C) zinc acetate in NMP, and (D) zinc nitrate in NMP.

Beyond the precursor, the dispersion solvent also proved decisive for colloidal behavior. When dispersed in water (Figure 3A and B), ZnO NPs showed significantly larger hydrodynamic diameters, ranging from 246 to 1470 nm, and in some cases secondary populations of larger particles, indicating aggregation. Conversely, dispersions in NMP (Figure 3C and D) resulted in smaller and more homogeneous size distributions, reflecting greater stability and reduced aggregation. These results highlight the importance of solvent selection in controlling the dispersion and colloidal stability of ZnO NPs, a key factor for their application.^48,49^

A complementary perspective comes from morphological analysis by SEM (Figure 4). The images revealed well-defined crystalline cores with diameters between 49 and 131 nm, in agreement with the range typically reported in the literature (20–100 nm).^1,2^ This direct visualization contrasts with DLS results, which measure the hydrodynamic diameter, including the solid core, solvation layer, and particle aggregates in suspension. Due to their high surface energy, ZnO nanoparticles tend to aggregate in liquid media as a means of minimizing surface free energy. In DLS measurements, these aggregates are treated as single scattering entities, leading to an overestimation of the average particle size, as widely reported in the literature.^50^ This aggregation behavior is characteristic of metal oxide nanoparticles and does not contradict the nanoscale primary particle sizes observed by SEM.

**Figure 4:**
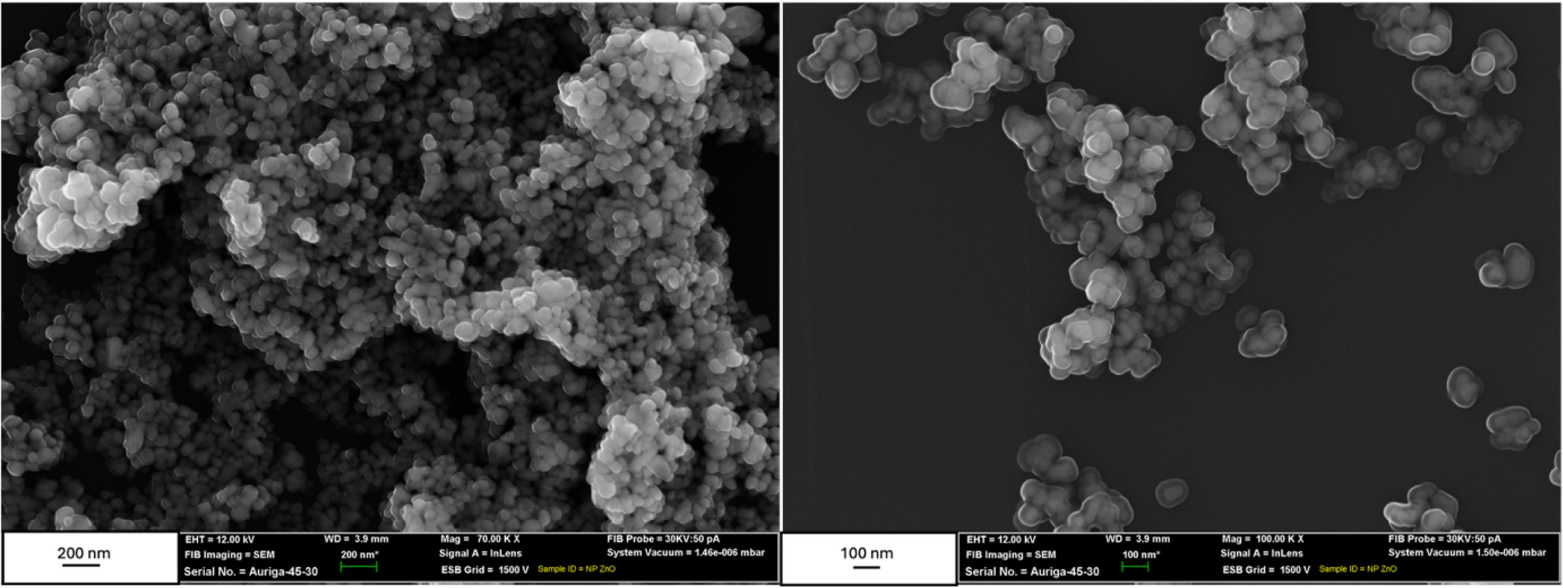
SEM images of the synthesized ZnO nanoparticles.

As expected, the particle sizes determined by DLS were considerably larger than those obtained by SEM, reflecting the fundamental methodological differences between these techniques rather than inconsistencies in nanoparticle synthesis. Similar discrepancies between particle sizes measured by DLS and electron microscopy techniques have been reported by Wang et al.^51^ and Winiarska et al.,^52^ reinforcing the importance of interpreting DLS data in conjunction with complementary morphological analyses to achieve a reliable and comprehensive characterization of nanoparticle systems.^48^ In the context of membrane fabrication, this behavior highlights the relevance of solvent–particle–polymer interactions, as aggregation in suspension may influence particle distribution and pore formation during casting.

The colloidal stability of the suspensions was further evaluated through zeta potential measurements (Figure 5). Despite the tendency toward aggregation in aqueous media, some samples-maintained zeta potential values above +30 mV, which is indicative of satisfactory colloidal stability. This finding demonstrates that the synthesis route successfully balanced particle size and surface charge, both essential parameters for environmental and biomedical applications.

**Figure 5:**
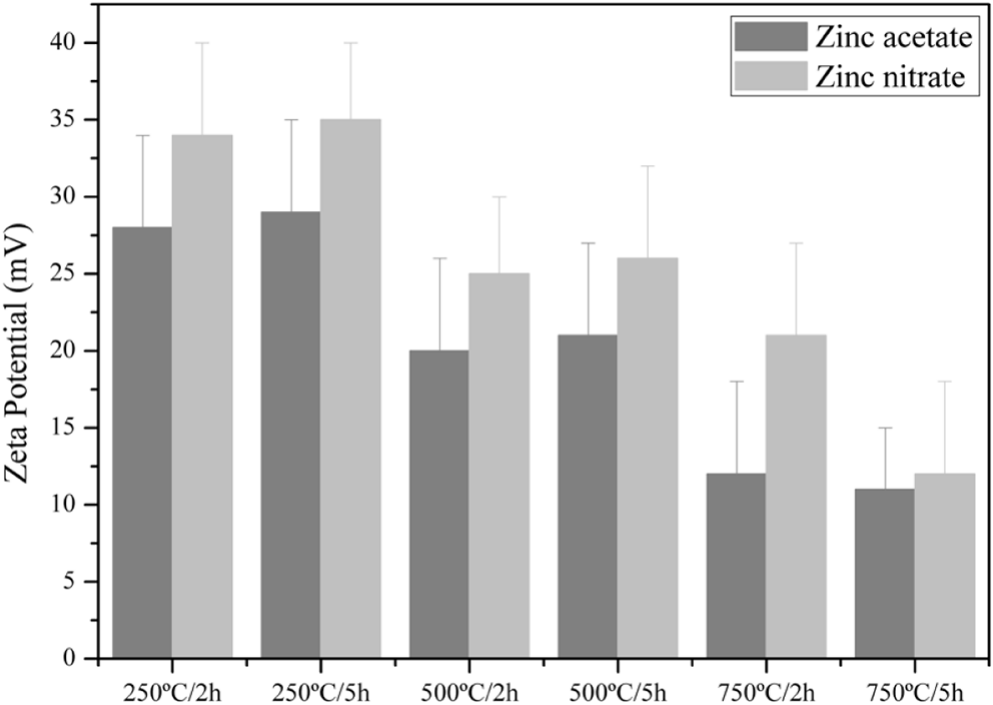
Zeta potential of ZnO nanoparticles dispersed in water, synthesized from different precursors (zinc acetate and zinc nitrate) and calcination parameters (2 h and 5 h; 250, 500, and 750 °C).

Based on the results obtained, the most suitable ZnO nanoparticles for incorporation into a PES-ZnO membrane matrix were those synthesized from zinc acetate and calcined at 250 °C for 5 h. This selection was guided by their smaller size, narrower and more homogeneous size distribution, and satisfactory colloidal stability in aqueous medium, combined with their excellent dispersion in solvent. These characteristics are critical for the membrane fabrication process, in which nanoparticles must first be well dispersed in the solvent before the polymer is added, thereby ensuring a uniform distribution of ZnO throughout the polymer matrix. Overall, the findings of this study are consistent with those reported by Pourrahimi et al.,^47^ Gatou et al.^49^ and José & Shinde,^48^ validating the effectiveness of the synthesis route in producing ZnO nanoparticles with nanoscale dimensions and satisfactory colloidal stability.^1–3^

### 3.2 Membrane Characterization

Figure 6 presents the scanning electron microscopy (SEM) images of the synthesized membranes’ cross sections. All synthesized membranes exhibited an asymmetric structure, consisting of a dense selective skin layer supported by a porous substructure, a typical feature of membranes fabricated by the NIPS method. This structural configuration enables a favorable balance between high selectivity, attributed to the top layer, and high permeability, provided by the porous support. The unmodified PES membrane (Figure 6A) displayed a substructure predominantly of the finger-like type, composed of elongated macrovoids oriented perpendicular to the membrane surface. This morphology is associated with a rapid precipitation process, resulting from intense exchange between solvent and nonsolvent, which triggers a binodal demixing via nucleation and growth, leading to the formation of vertical channels with low polymer density.^53^

**Figure 6:**
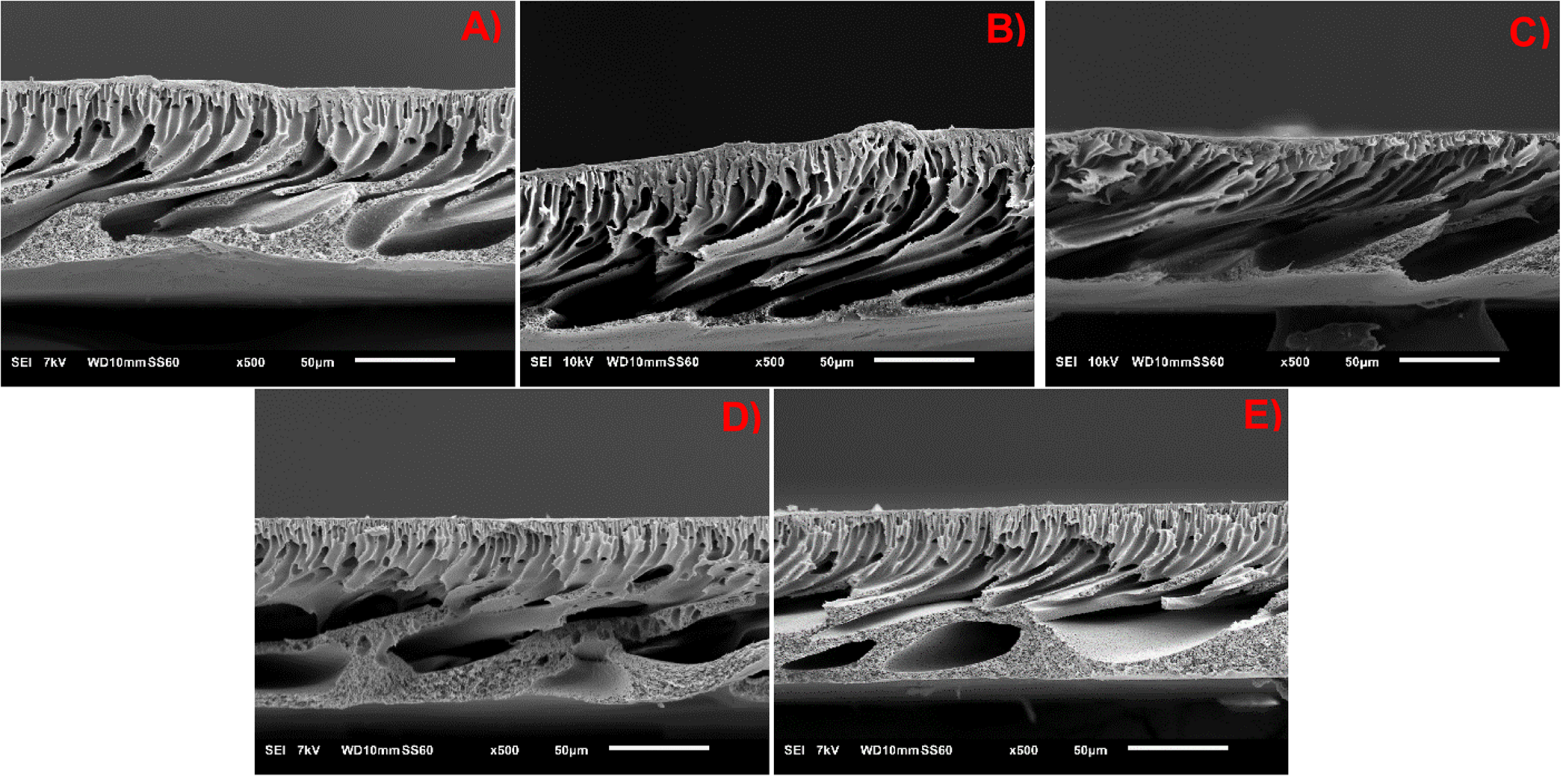
SEM images of the synthesized membranes. A) PES; B) ZnO-0.25%; C) ZnO-0.50%; D) ZnO-0.75%; E) ZnO-1.00%.

Upon incorporation of ZnO into the polymer matrix, a progressive change in the morphology of the substructure was observed. In the membranes containing 0.25% and 0.50% ZnO by weight (Figure 6B and C), macrovoids were still present but became shorter and less frequent. From 0.75% ZnO onward (Figure 6D), the internal structure exhibited mixed characteristics, with a noticeable reduction in macrovoid content and an increase in sponge-like regions. At 1.00% ZnO (Figure 6E), the substructure became predominantly sponge-like, consisting of small, spherical, and interconnected pores. This morphological transition can be attributed to two main factors. First, the increasing ZnO concentration can be attributed to a possible increase in the viscosity of the casting solution, which would hinder nonsolvent diffusion into the polymer matrix and delay the phase separation. As a result, a slower demixing kinetics is established, favoring the formation of sponge-like structures. Second, ZnO nanoparticles may interfere with the thermodynamics of the ternary system (polymer/solvent/nonsolvent), altering the binodal boundary and promoting phase separation under more homogeneous conditions, as discussed by Porter.^53^

These findings are consistent with the literature, which reports that the addition of nanoparticles alters membrane morphology, especially due to their affinity with the nonsolvent (water) and their influence on the thermodynamic stability of the casting solution.^54–57^ According to Fang et al.,^58^ the formation of macrovoids requires a high influx of nonsolvent into the casting film, penetrating deeply and inducing rapid precipitation along vertical channels. The propagation of these macrovoids may be enhanced by structural weaknesses introduced by additives or polymer matrix heterogeneities.^56^

Additionally, ZnO nanoparticles may influence phase redistribution due to their high interfacial energy. At higher concentrations, they tend to migrate or leach partially during coagulation, potentially generating regions with higher porosity in the substructure.^59^ Furthermore, the lower part of the membrane, less exposed to the initial solvent/nonsolvent exchange, tends to develop larger and more interconnected pores, forming sponge-like regions,^60^ as also observed in Figure 6D and E.

Table 1 presents zeta potential, contact angle, porosity, mean pore radius (rm), and hydraulic permeability for the pristine PES membrane and the ZnO-modified ones. The zeta potential became progressively more negative with increasing ZnO loading, from −13.9 ± 0.1 mV (PES) to −32.7 ± 0.2 mV at 1.00% ZnO, which is consistent with the exposure of negatively charged surface hydroxyls on ZnO that raise the surface charge density.^61^ A more negative zeta potential is advantageous for repelling anionic organic foulants (e.g., humic substances and proteins) and can therefore mitigate adsorption-driven fouling.^62^ In contrast, the static water contact angle varied only slightly (50.3° to 53.3°), showing no monotonic trend with ZnO.

**Table 1:** Physicochemical Properties and Performance Parameters of PES Membranes and Membranes Modified with Different ZnO Concentrations

Membrane	Zeta potential (mV)	Contact angle (°)	Porosity (ε%)	r_m_ (μm)	Permeability (L m^–2^h^–1^bar^–1^)
PES	–13.9 ± 0.1	51.5 ± 1.8	67.0 ± 5.6	0.029	158 ± 29
PES-ZnO_0.25_	–26.0 ± 0.3	53.3 ± 1.5	71.4 ± 2.5	0.033	301 ± 62
PES-ZnO_0.50_	–31.4 ± 0.4	51.1 ± 1.1	53.5 ± 3.3	0.049	367 ± 36
PES-ZnO_0.75_	–28.1 ± 0.2	50.3 ± 1.5	71.1 ± 2.3	0.043	518 ± 79
PES-ZnO_1.0_	–32.7 ± 0.2	50.3 ± 1.8	69.9 ± 4.6	0.027	186 ± 6

Although the incorporation of ZnO did not lead to significant changes in the static water contact angle, a pronounced shift toward more negative zeta potential was observed. This apparent decoupling reflects the fact that these parameters probe different interfacial phenomena. The contact angle represents macroscopic wettability measured at the air–water interface under static conditions, whereas zeta potential characterizes the effective surface charge within the hydrated interface under aqueous conditions relevant to filtration. Negatively charged functional groups introduced by ZnO, particularly surface hydroxyls, can reside within pore walls or subsurface regions and significantly modify the electrical double layer without substantially affecting the outermost surface wettability.^63^ Additionally, partial coverage of nanoparticles by the polymer skin layer, along with local roughness/heterogeneity, can mask modest chemical changes in static contact-angle measurements.^64,65^ Under filtration conditions, this enhanced negative surface charge increases electrostatic repulsion between the membrane and negatively charged organic foulants, reducing adsorption strength and promoting the formation of loosely attached, more reversible deposits. Consequently, antifouling performance can be improved through electrostatic effects even when changes in macroscopic hydrophilicity are minimal.^66^ Overall, while ZnO incorporation did not produce a pronounced change in static wettability, it substantially increased interfacial charge, an effect that is mechanistically consistent with reduced foulant adhesion even at similar contact angles.^67,68^

Regarding surface roughness (S_a_), the variations among the formulations were also not pronounced (Figure 7). The PES membrane exhibited a roughness of 45.1 ± 11.6 nm, while the ZnO-modified membranes showed slightly lower and more consistent values, with a notable result for the 0.75% ZnO formulation (31.4 ± 4.8 nm). Although these differences are modest, the data suggest a trend toward surface smoothing with the incorporation of ZnO, possibly due to better nanoparticle dispersion and reduced heterogeneities in the polymer matrix. AFM images support this interpretation, showing a reduction in the amplitude of surface peaks and valleys in the modified membranes, especially in the 0.75% ZnO sample, reinforcing the hypothesis of a more uniform surface topography.

**Figure 7:**
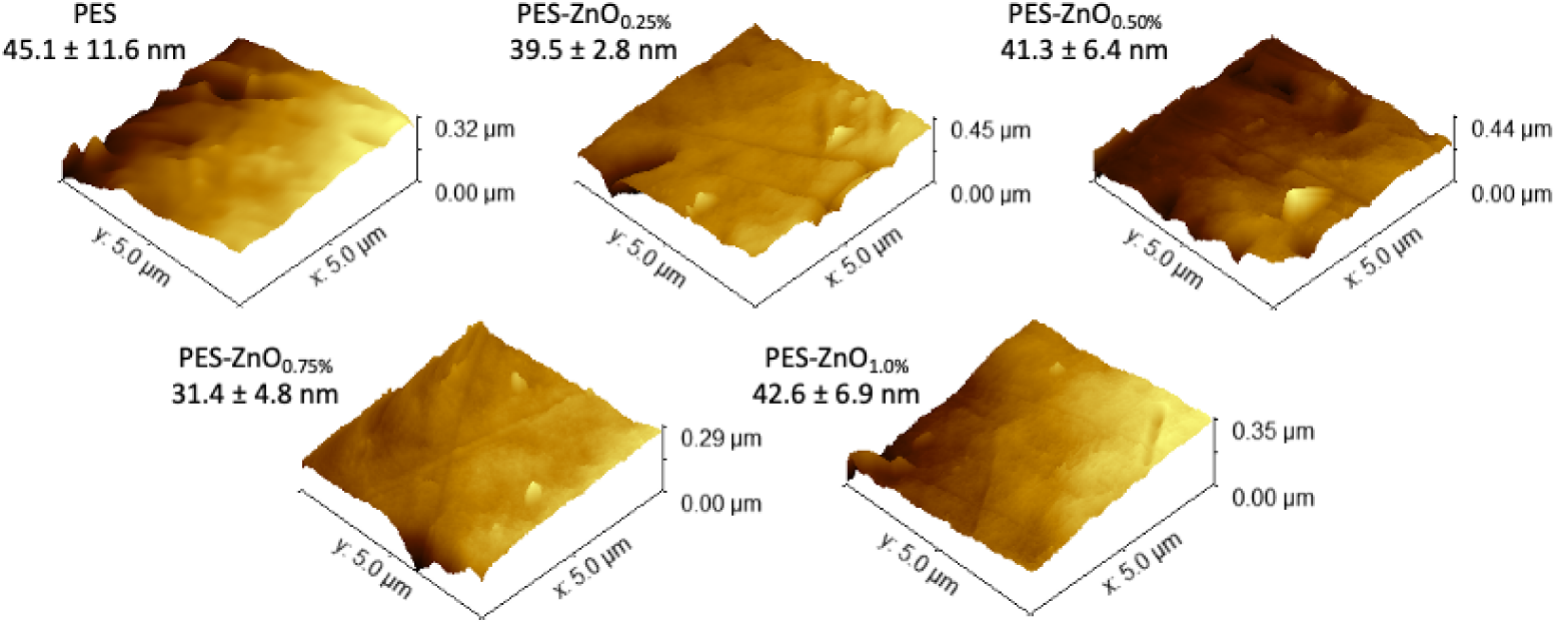
Surface topography images obtained by Atomic Force Microscopy (AFM) in tapping mode of PES membranes and membranes modified with different concentrations of ZnO.

The apparent porosity of the membranes showed a trend of increasing with the addition of ZnO, especially at concentrations of 0.25% and 0.75%, which exhibited values higher than those of the unmodified PES membrane. On the other hand, the formulation containing 0.50% ZnO presented slightly lower porosity. These fluctuations suggest that the presence of ZnO affects membrane morphology in a nonlinear manner, which may be associated with competing effects during the phase inversion process, such as increased viscosity of the casting solution and thermodynamic instability induced by the nanoparticles.^31,59^

The mean pore radius (r_m_) followed a similar trend, with higher values observed for the PES-ZnO_0.25_ (0.033 μm), PES-ZnO_0.50_ (0.049 μm) and PES-ZnO_0.75_ (0.043 μm), followed by a decrease at higher concentrations (0.027 μm for PES-ZnO_1.00_). The hydraulic permeability of the membranes largely reflects the trend observed for the mean pore radius. The unmodified PES membrane exhibited the lowest permeability value (158 L m^–2^ h^–1^ bar^–1^), while the membranes containing 0.25%, 0.50%, and 0.75% ZnO showed progressive increases, reaching a maximum of 518 L m^–2^ h^–1^ bar^–1^ for the 0.75% formulation. This significant improvement can be attributed to a combination of factors, including enhanced internal pore connectivity, the presence of transport-favorable pore structures, and possible surface smoothing. Even though the PES-ZnO_0.75_ membrane had a lower mean pore radius than the 0.50% formulation, it exhibited superior hydraulic performance, suggesting that, in addition to pore size, pore distribution and interconnectivity also played crucial roles. In contrast, the membrane PES-ZnO_1.00_ showed a slight reduction in permeability (186 L m^–2^ h^–1^ bar^–1^) compared to the PES-ZnO_0.75_ formulation, despite maintaining relatively high porosity. This behavior may be related to the formation of smaller pores and increased structural tortuosity, possibly exacerbated by nanoparticle agglomeration or densification of the skin layer. As highlighted in previous studies, there appears to be an optimal ZnO concentration for enhancing hydraulic performance, while excessive nanoparticle loading may compromise the desirable membrane structure.

### 3.3 Filterability and Antifouling Performance

Figure 8 shows the permeate flux profiles over time during the surface water filtration tests under constant pressure mode. It can be observed that both the pattern and the intensity of flux decline varied substantially among the different membrane formulations. The membrane PES-ZnO_0.25_ exhibited the best performance, with a much smoother and more gradual flux decline, consistently maintaining values significantly above the others. This behavior suggests lower initial particle adhesion and a slower, more diffuse development of the cake layer. According to Yoon,^69^ in systems operating under constant pressure, the initially high flux favors particle deposition, as the back-transport forces are not always sufficient to counteract the convective transport of contaminants toward the membrane surface. However, when membrane–foulant interactions are minimized, as observed in membranes with more negative surface charge (higher zeta potential), the initial adsorption of material and onset of cake formation can be delayed, as observed for PES-ZnO_0.25_ and PES-ZnO_0.50_. The PES-ZnO_0.50_ showed a more pronounced initial decline, possibly due to its higher porosity and more accessible pores, which may have facilitated the initial entry of particles, but the flux quickly stabilized at a high level.

**Figure 8:**
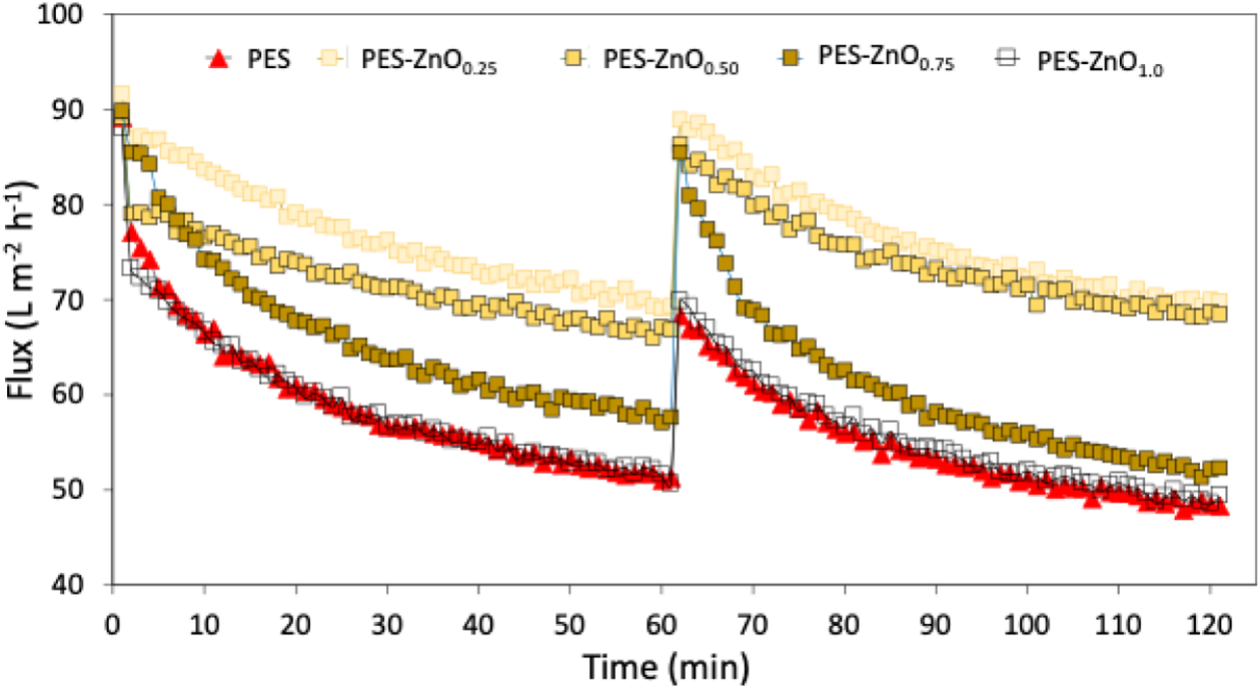
Permeate flux over time during two constant-pressure filtration cycles with surface water for PES and ZnO-modified membranes. Curves correspond to representative runs for each membrane formulation. Reproducibility was assessed through duplicate filtration cycles.

In contrast, PES-ZnO_0.75_, despite exhibiting the highest hydraulic permeability under ultrapure water tests, did not maintain the same performance during surface water filtration, indicating greater susceptibility to fouling compared to the membranes with 0.25% and 0.50% ZnO. For the PES and PES-ZnO_1.0_ membranes, flux declined more sharply and showed no signs of stabilization over time. This behavior indicates rapid formation and compaction of the cake layer and suggests that, under identical hydrodynamic conditions, the weaker foulant–membrane interactions in PES-ZnO_0.25_ and PES-ZnO_0.50_ likely facilitated particle back-transport to the bulk solution, reducing accumulation, whereas PES and PES-ZnO_1.0_ retained foulants more strongly, promoting faster fouling and flux decline.

Fouling at the beginning of filtration is mainly associated with hydrophobic, electrostatic, or covalent adsorption of contaminants on the membrane surface and within the pore walls. These compounds, once adhered, may constrict or block the membrane pores and negatively affect permeate flux.^70^ Therefore, the addition of ZnO nanoparticles reduced initial adsorption-related fouling in the modified membranes (up to 0.75% ZnO), when compared to unmodified PES membranes, considering that all filtration tests were initiated at the same flux and thus exposed to similar contaminant loads. According to Xu et al.,^70^ membrane–foulant interactions are governed by surface charge, roughness, pore size, and spatial distribution; thus, the combined effects of a more negative surface charge and suitable pore size likely promoted size exclusion and electrostatic repulsion in PES-ZnO_0.25_ and PES-ZnO_0.50_. At the end of the second filtration cycle, the permeate fluxes were 48, 70, 68, 52, and 49 L m^–2^ h^–1^, corresponding to permeabilities of 39, 107, 105, 80, and 37 L m^–2^ h^–1^ bar^–1^ for the PES, PES-ZnO_0.25_, PES-ZnO_0.50_, PES-ZnO_0.75_, and PES-ZnO_1.0_ membranes, respectively, clearly indicating best sustained performance for 0.25–0.50% ZnO membranes, the membrane with 0.75% ZnO had an intermediate performance, while the one with 1.0% ZnO performed as the PES.

As shown in Figure 9A, the total fouling resistance was significantly higher for the PES and PES-ZnO_1.0_ membranes, reaching approximately 3.4 × 10^12^ m^–1^ in the second cycle. This result is consistent with the sharp flux decline observed for these formulations. In contrast, the membranes containing 0.25% and 0.50% ZnO exhibited the lowest total resistance values (5.4 × 10^11^ m^–1^ in both cases), representing a reduction of over 80% compared to PES. This performance reinforces the effectiveness of these compositions in fouling mitigation and aligns with the flux results, which showed lower loss over time. Additionally, the fouling resistance profile also varied. For the membranes with 0.25% and 0.50% ZnO, in addition to the substantial reduction in total resistance, most of the fouling was reversible (above 74% in both cycles), indicating that the deposited material was weakly attached to the membrane and could be easily removed by simple water rinsing. This pattern was maintained in the second cycle, suggesting good stability and potential use of the membrane.

**Figure 9:**
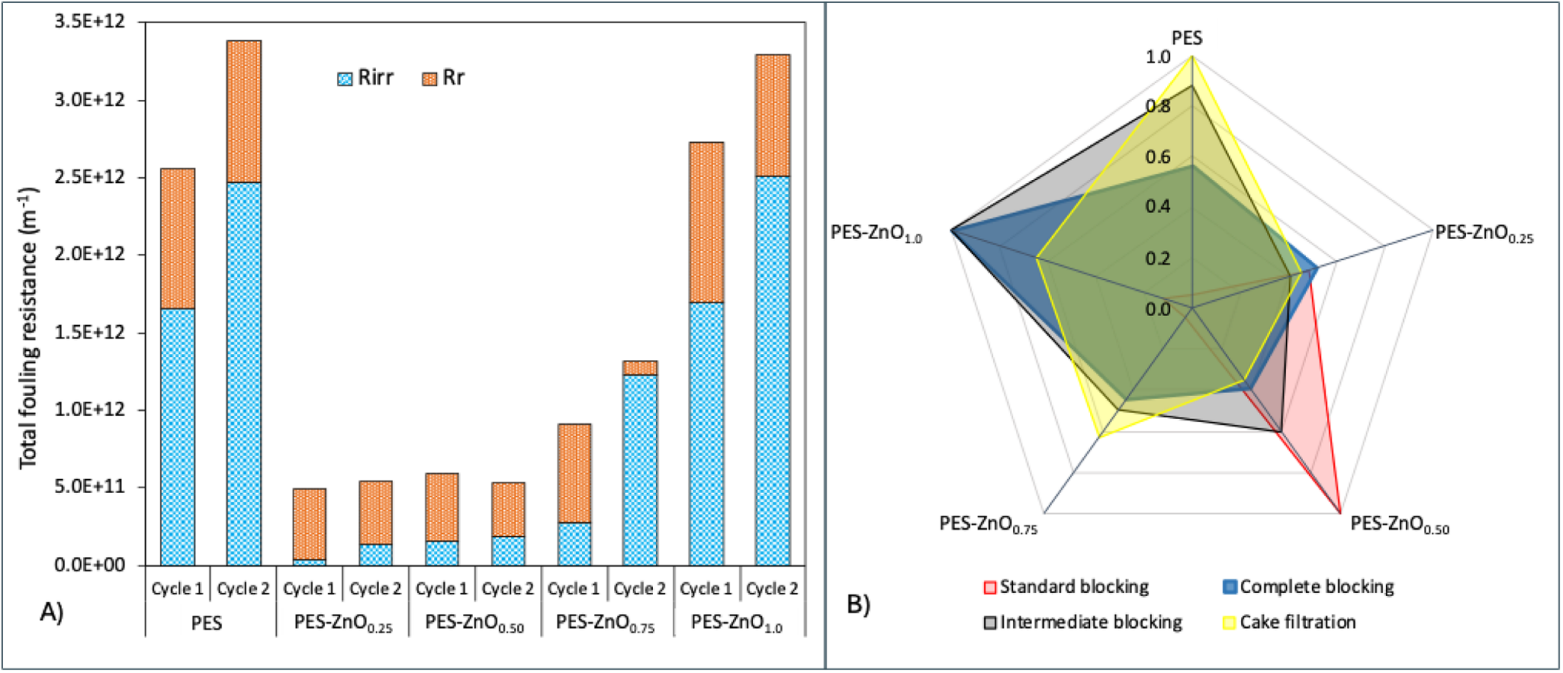
Fouling resistance for each membrane after two filtration cycles (A) and normalized inverse mean squared error (1/MSE) obtained from Hermia model fitting (B), where values closer to 1 indicate a better agreement between experimental flux data and the corresponding fouling mechanism.

The PES-ZnO_0.75_ membrane, despite exhibiting the highest hydraulic permeability under ultrapure water conditions, showed a marked increase in irreversible resistance during filtration of real surface water, rising from 2.8 × 10^11^ to 1.2 × 10^12^ m^–1^ in the second cycle and accounting for approximately 60% of the total resistance. This behavior indicates that the higher permeability was associated with a more open and accessible pore structure, which facilitated the penetration and retention of organic matter within the pores rather than forming a loosely attached surface layer. As a result, foulant–membrane interactions became stronger, leading to pore blocking and reduced flux recovery. These findings highlight that high pure water permeability alone is not a reliable predictor of membrane performance under realistic conditions, where pore architecture and foulant interactions play a dominant role in governing fouling resistance and rejection efficiency.

The membrane PES-ZnO_0.75_, despite exhibiting the highest hydraulic permeability in ultrapure water tests, showed a significant increase in irreversible resistance in the second cycle (from 2.8 × 10^11^ to 1.2 × 10^12^ m^–1^), accounting for 60% of the total resistance. This behavior indicates that the higher permeability was associated with a more open and accessible pore structure, which facilitated the penetration and retention of organic matter within the pores rather than forming a loosely attached surface layer. As a result, foulant–membrane interactions became stronger, leading to pore blocking and reduced flux recovery. These findings highlight that high pure water permeability alone is not a reliable predictor of membrane performance under realistic conditions, where pore architecture and foulant interactions play a dominant role in governing fouling resistance and rejection efficiency. Meanwhile, the membrane containing 1.00% ZnO displayed a resistance profile like that of the unmodified PES membrane, with high total resistance and predominance of irreversible fouling in both cycles. These findings reinforce that excessive nanoparticle loading can compromise membrane structure and intensify irreversible deposition mechanisms.

The analysis of proteins and humic substances (UV_254_) extracted from the membrane surface with deionized water after the filtration assays is presented in Table 2. Higher amounts of proteins and UV_254_ extracted by simple water rinsing indicate that a larger fraction of the deposited material was weakly attached and therefore reversible, whereas lower extracted values are associated with stronger foulant adhesion and a higher contribution of irreversible fouling. Distinct patterns of foulant removal can be observed, especially between PES and PES-ZnO_0.25_. PES showed the lowest extracted values (2.13 mg L^–1^ protein; 0.066 cm^–1^ UV_254_), yet high total/irreversible resistance, suggesting that a large portion of the deposit was irreversible, strongly adhered, and therefore little affected by simple water extraction. By contrast, PES-ZnO_0.25_ presented the highest extracted amounts (5.11 mg L^–1^ protein; 0.112 cm^–1^ UV_254_), i.e., ∼140% more protein and ∼70% more UV_254_ than PES, consistent with a weakly adhered, mostly reversible surface layer and the high flux recovery observed. For the other formulations (0.50–1.00% ZnO), the protein values were very similar (≈3.0–3.4 mg L^–1^), with no clear trend. For UV_254_, the differences were also small: a slight reduction for PES-ZnO0.50 (0.05 ± 0.001 cm^–1^) and values close to those of PES and ZnO_0.75_–1.00% (0.07–0.06 cm^–1^).

**Table 2:** Quantification of Proteins and Absorbance at 254 nm (UV_254_) Extracted from the Membrane Surface after Filtration Tests, Using Rinsing with Deionized Water^a^

Membrane	Protein (mg L^–1^)	UV_254_ (cm^–1^)
PES	2.13 ± 0.14	0.066 ± 0.001
PES-ZnO_0.25_	5.11 ± 0.14	0.112 ± 0.001
PES-ZnO_0.50_	2.94 ± 0.14	0.054 ± 0.001
PES-ZnO_0.75_	3.34 ± 0.14	0.071 ± 0.001
PES-ZnO_1.0_	3.42 ± 0.14	0.056 ± 0.001

a Values are presented as mean ± standard deviation.

When integrated with the hydraulic behavior, the larger mass extracted for PES-ZnO_0.25_ is compatible with predominantly superficial and reversible fouling, whereas the low removal from the PES membrane suggests deposits that are not easily accessed by water rinsing. In this sense, the 0.25% ZnO formulation likely benefited from a more negative surface charge and a favorable porous architecture, facilitating electrostatic repulsion and the formation of less compact layers, which explains its superior flux recovery. Because the 0.50%, 0.75%, and 1.00% membranes exhibited similar extracted amounts, the performance differences among them arise less from how much was deposited and more from where and how the material attached: in ZnO–0.50%, the lower UV_254_ suggests weaker adhesion of humic substances (electrostatic repulsion plus pore architecture), supporting good recoverability; in ZnO–0.75% and 1.00%, despite similar extractions, the larger irreversible fraction likely indicates pore blocking/compaction that is not captured by aqueous extraction.

In addition to the flux and fouling resistance analyses, the Hermia models (Figure 9B) were applied to evaluate the predominant deposition mechanisms during the filtration of surface water, considering complete blocking, standard blocking, intermediate blocking, and cake filtration mechanisms. The PES membrane showed the best fit for the cake layer formation mechanism (1.0), followed by intermediate blocking (0.88), suggesting that the flux decline was mainly associated with surface particle deposition combined with some progressive penetration into the polymer matrix. The addition of ZnO altered this pattern. For the PES-ZnO_0.25_ membrane, no single mechanism stood out, with similar fit values for cake filtration (0.46), standard blocking (0.48), and complete blocking (0.52). This absence of a predominant mechanism suggests the simultaneous occurrence of multiple fouling processes.^71^ Rather than indicating a limitation of the Hermia model, this result reflects the coexistence of multiple fouling mechanisms acting simultaneously. Such behavior is consistent with filtration of real surface water, which contains a heterogeneous mixture of organic matter, colloids, and dissolved compounds, promoting sequential and overlapping fouling processes instead of a single dominant regime. In this case, the absence of a prevailing mechanism is also coherent with the low total resistance and high fraction of reversible fouling observed, suggesting weak foulant–membrane interactions and a dynamic balance between surface deposition and partial pore intrusion.

For the PES-ZnO_0.50_ membrane, a shift in the predominant mechanism was observed, with a high fit for the standard blocking model (1.00). This result indicates that smaller particles partially penetrated the pores, causing progressive obstruction without the dominant formation of a surface cake layer. This behavior is consistent with the higher porosity and larger average pore size of this membrane, as well as the good performance observed in the filtration tests, characterized by high permeate flux and moderate fouling resistance. Previous studies, such as Choobar et al.,^72^ have also reported the predominance of standard blocking during intermediate filtration stages, especially when fine particles gradually accumulate within the pore channels.

The membrane containing 0.75% ZnO exhibited intermediate fit values for all tested fouling mechanisms (e.g., 0.63 for cake filtration, 0.50 for intermediate blocking, and 0.45 for complete blocking), with no clear identification of a predominant mode. Similar to what was observed for the PES-ZnO_0.25_ membrane, this pattern indicates the coexistence of simultaneous deposition and penetration processes. On the other hand, the significant accumulation of irreversible fouling may be related to the greater internal structural heterogeneity and larger average pore radius of this membrane, as well as its lower zeta potential, which favors particle penetration into the pores and the establishment of stronger electrostatic interactions with the membrane matrix. In contrast, the membrane with 1.00% ZnO showed the highest fit values for the complete blocking (1.00) and intermediate blocking (1.00) mechanisms, indicating strong pore blockage in the early stages of filtration followed by progressive particle penetration into the membrane structure. This behavior is typical of membranes with a dense structure and small, poorly interconnected pores, which could explain the high degree of irreversible fouling observed even in the presence of a strongly negative surface charge.

The results obtained in this study are consistent with the literature demonstrating the positive effect of ZnO incorporation on fouling resistance in polymeric membranes, especially PES. Previous studies have reported that moderate ZnO concentrations (∼0.5–1.0%) promote increased hydrophilicity, improved porous structure, and significant reduction of irreversible fouling. For example, Li et al.^73^ observed a substantial increase in the permeability of PES-ZnO membranes (from 46.4 to 365.8 L·m^–2^·h^–1^) and a reduction in the irreversible fouling fraction to about 17%, with flux recovery close to 80% after simple water washing. Similarly, Ahmad et al.^74^ reported greater flux and rejection maintenance in ZnO-PES membranes, as well as a significant reduction in fouling during humic acid filtration. In systems with real water, a recent study with reservoir water showed that a PES membrane containing 0.5% ZnO achieved the best performance among all tested formulations, combining high pure water flux (∼427 L·m^–2^·h^–1^) with higher fouling resistance than membranes without ZnO or with 1% ZnO. The optimal performance in this formulation was attributed to increased hydrophilicity and the absence of nanoparticle agglomeration at moderate concentrations. In line with these findings, the present study demonstrated that membranes modified with 0.25% and 0.50% ZnO exhibited the lowest total fouling resistances, with a predominance of the reversible fraction (above 74%) and high flux recovery rates (96.8% and 93.9%, respectively), indicating performance equivalent to or superior to that observed in previous studies, even when subjected to raw water filtration.

### 3.4 Membrane Performance in Pollutant Removal and Practical Applications in Drinking Water Treatment

The characteristics of the source water, as well as the concentrations of turbidity, color, UV_254_ absorbance, and total organic carbon (TOC) in the permeates obtained from each membrane, are presented in Table 3. All membranes were highly efficient in turbidity removal, reducing the initial value of 2.22 ± 0.14 NTU to less than 0.1 NTU, regardless of the ZnO content. However, more significant differences were observed for the other parameters, especially for color and organic matter removal, which are generally more sensitive to the surface and structural properties of the membranes, since the rejection of dissolved organic matter is governed by a combination of intrinsic membrane properties, such as pore size distribution and surface charge, and by the development of a fouling layer during operation, which may act as a secondary and dynamic selective barrier.^75^

**Table 3:** Raw Water and Permeate Quality Parameters after Filtration with PES and PES-ZnO Membranes

Sample		Turbidity (NTU)	Color (Pt–Co)	UV_254_ (cm^–1^)	TOC (mg L^–1^)
Raw water		2.22 ± 0.14	28.1 ± 3.0	1.02 ± 0.21	7.4 ± 1.6
Permeate	PES	0.08 ± 0.02	14.0 ± 0.6	0.71 ± 0.07	2.8 ± 0.2
	PES-ZnO_0.25_	0.02 ± 0.01	14.7 ± 1.2	0.76 ± 0.04	2.3 ± 0.3
	PES-ZnO_0.50_	0.04 ± 0.01	7.0 ± 1.0	0.79 ± 0.02	2.7 ± 0.3
	PES-ZnO_0.75_	0.03 ± 0.02	21.7 ± 1.5	0.85 ± 0.03	5.6 ± 0.4
	PES-ZnO_1.0_	0.03 ± 0.01	14.7 ± 1.1	0.68 ± 0.03	2.1 ± 0.9

Regarding apparent color, a greater variability among the formulations was observed. The control PES membrane reduced color from 28.1 ± 3.0 to 14.0 ± 0.6 Pt–Co, corresponding to a 50.2% removal. The PES-ZnO_0.25_ and PES-ZnO_1.0_ membranes showed similar performance, with removals of 47.7% and 47.6%, respectively. In contrast, the PES-ZnO_0.50_ membrane achieved the highest removal efficiency, reaching 75.1% (final color of 7.0 ± 1.0 Pt–Co). Conversely, the PES-ZnO_0.75_ formulation exhibited the lowest performance, with only 22.8% color removal and a remaining color concentration of 21.7 ± 1.5  Pt–Co.

The UV_254_ absorbance results, associated with the presence of humic substances, also revealed relevant differences. The raw water presented a mean value of 1.02 ± 0.21 cm^–1^, which was reduced to 0.71 ± 0.07 cm^–1^ by the PES membrane (30.4% removal). Similar performance was observed for the membranes modified with 0.25%, 0.50%, and 1.00% ZnO, which produced permeates with UV_254_ values of 0.76 ± 0.04 cm^–1^, 0.79 ± 0.02 cm^–1^, and 0.68 ± 0.03 cm^–1^, respectively. For TOC, the results followed a trend like that of UV_254_. The PES membrane reduced TOC from 7.4 ± 1.0 to 2.8 ± 0.2 mg L^–1^ (62.2% removal), while the PES-ZnO_0.25_, PES-ZnO_0.50_, and PES-ZnO_1.0_ membranes produced permeates with concentrations ranging from 2.3 ± 0.3 to 2.7 ± 0.3 mg L^–1^. On the other hand, the PES-ZnO_0.75_ membrane showed lower efficiency in removing dissolved organic carbon, with a final TOC concentration of 5.6 ± 0.4 mg L^–1^, nearly double that of the control PES membrane.

The membrane containing 0.75% ZnO showed the lowest rejection capacity among all tested formulations, removing only 22.8% of color, 16.7% of UV_254_ absorbance, and 24.3% of TOC. These values indicate a lower performance not only compared to membranes with lower ZnO content, but also in relation to the unmodified PES control membrane. On the other hand, this same formulation exhibited the highest hydraulic permeability in tests with deionized water (518 L m^–2^ h^–1^ bar^–1^), a result attributed to its high porosity (71.1%) and larger mean pore radius (0.043 μm). Taken together, these data highlight the trade-off between permeability and rejection, commonly observed in the synthesis of mixed polymeric membranes. Although increased porosity enhances water transport, it also tends to reduce solute residence time at the membrane interface and hinder mechanisms such as size exclusion and electrostatic repulsion. In this case, the low rejection observed for the PES-ZnO_0.75_ membrane suggests that intrinsic membrane properties, particularly the larger effective pore size and reduced solute–membrane interaction time, dominated over any potential contribution of the fouling layer to pollutant rejection. In the case of the PES-ZnO_0.75_ membrane, this imbalance resulted in color (21.7 Pt–Co) and TOC (5.6 mg L^–1^) concentrations in the permeate that exceed the limits established by Brazilian regulations for drinking water (15 Pt–Co for color and, indirectly, 5.0 mg L^–1^ for TOC as a control parameter in waters with turbidity ≤2 NTU), as defined in Ordinance GM/MS No. 888, of May 4, 2021. Furthermore, the permeate color also surpasses the aesthetic guideline value recommended by the World Health Organization (WHO), which advises color levels below 15 Pt–Co to ensure acceptable drinking water quality and effective treatment performance.^76^

Overall, the results obtained for the 0.25% ZnO formulation stand out for their balance between hydraulic performance and pollutant rejection capacity, along with remarkable fouling resistance. Therefore, for the PES-ZnO_0.25_ membrane, rejection performance appears to result from a synergistic effect between favorable intrinsic membrane properties and the formation of a mild, reversible fouling layer acting as a dynamic filtration barrier. This combination of properties, achieved with an extremely low nanoparticle loading, reinforces the technical and practical potential of this composition for real-world applications. Considering that many surface water sources in urban areas exhibit low turbidity but high concentrations of dissolved organic matter, membranes with this profile may represent a viable and efficient alternative, including from an economic and environmental standpoint, for the development of commercial ultrafiltration membranes aimed at treating water for public supply.

## 4 Conclusions

Incorporating ZnO into PES ultrafiltration membranes improved fouling control and hydraulic performance when treating real surface water. The most effective range was 0.25–0.50 wt % ZnO, which combined high sustained flux with low total resistance (∼5.4 × 10^11^ m^–1^), a predominance of reversible fouling (>74%), and excellent flux recovery (96.8–93.9%). Foulant extraction confirmed that the 0.25% ZnO membrane formed loosely adhered deposits. By contrast, the 0.75% ZnO membrane, despite its highest pure-water permeability, showed increased irreversible resistance and lower pollutant rejection, illustrating a trade-off between permeability and selectivity. The 1.00% ZnO membrane resembled unmodified PES, with high total resistance and stronger foulant retention. In summary, these results indicate that low ZnO loadings tune surface charge and pore architecture to favor electrostatic repulsion and less compact deposit formation, yielding superior antifouling behavior under realistic conditions. Practically, PES-ZnO membranes with 0.25–0.50% ZnO are promising candidates for drinking-water applications in organic-rich surface sources. Moreover, the use of such a low nanoparticle loading facilitates large-scale implementation, reducing both costs and the environmental risks associated with nanomaterials. Future work should address the long-term stability of ZnO within the membrane matrix, including potential nanoparticle leaching under prolonged operation and cleaning conditions, as well as the evaluation of cleaning strategies beyond simple water rinsing and scale-up in pilot-scale modules.
